# Rapid progression from croup to complete lung collapse: a case report of central airway obstruction by mucopurulent cast in influenza A (H3N2) and Pseudomonas aeruginosa coinfection in a toddler

**DOI:** 10.3389/fped.2026.1801970

**Published:** 2026-06-02

**Authors:** Xiumin Zhang, Fujing Xie, Jingcai Wang, Jing Zhao, Cuicui Guo

**Affiliations:** Department of Pediatric, Liaocheng People's Hospital, Liaocheng, Shandong Province, China

**Keywords:** croup, influenza A, lung collapse, mucous plug, pseudomonas aeruginosa

## Abstract

Croup is a common presentation in pediatric emergency settings, most often caused by viral infections. However, when influenza A virus co-occurs with *Pseudomonas aeruginosa* infection, it can lead to rare and lethal complications. We report a case of a 13-month-old boy who presented with fever and croup. The patient developed suffocation after 4.5 h of hoarseness and respiratory distress, with poor response to glucocorticoids therapy. Despite mechanical ventilation, respiratory failure persisted. Serial chest radiographs over a 7-h period revealed rapid progression to complete left lung atelectasis. Bronchoscopy identified complete obstruction of the left main bronchus by a purulent mucus plug, which was removed resulting in lung re-expansion. Etiological analysis confirmed co-infection with influenza A virus (H3N2) and *Pseudomonas aeruginosa*. The patient was treated with sensitive antibiotics and peramivir, and was discharged on day 9 with no recurrence of symptoms.

## 1 Introduction

Croup represents one of the most frequent etiologies of acute respiratory distress in young children. It exhibits a seasonal predilection for autumn and winter, with the highest incidence observed in children aged 6 months to 3 years, and a greater prevalence among males (1). The characteristic presentation includes a barking cough, often accompanied by stridor, hoarseness, and varying degrees of respiratory distress. These symptoms are typically preceded by 12–48 h of nonspecific upper respiratory prodromal features. The respiratory compromise is primarily attributed to upper airway obstruction resulting from virus-induced subglottic edema (2). Common viral pathogens include parainfluenza virus types 1 and 3, while other etiologic agents encompass influenza A virus (IAV) and influenza B virus (IBV), adenovirus, respiratory syncytial virus, and SARS-CoV-2 (2, 3). Bacterial infection is less frequently encountered. However, bacterial tracheitis should be considered in cases exhibiting rapid clinical deterioration or poor response to nebulized epinephrine therapy.

IAV, a member of the Orthomyxoviridae family, is a single-stranded negative-sense RNA virus. It constitutes a significant cause of morbidity and mortality in pediatric and geriatric populations, as well as individuals with chronic comorbidities or immunodeficiencies (4). The primary site of IAV infection and replication is the upper respiratory tract epithelium. In immunocompetent hosts, viral replication is generally confined to the upper airways (5). However, under conditions such as high viral inoculum, pre-existing respiratory conditions, young age, or altered immune status, the virus may disseminate inferiorly, triggering epithelial cell death, exaggerated inflammatory responses, and increased airway permeability (6, 7). Furthermore, commensal bacteria colonizing the nasopharynx may contribute to inflammation amplification and predispose to secondary bacterial infections. *Streptococcus pneumoniae*, *Haemophilus influenzae*, and *Staphylococcus aureus* are the most prevalent pathogens in secondary bacterial infections following influenza; nevertheless, *Pseudomonas aeruginosa (P. aeruginosa)* has been documented in severe cases (6, 8). The formation of mucopurulent casts represents a rare but severe complication of influenza pneumonia. A recent single-center study from Shenzhen Children's Hospital involving 321 children with influenza virus pneumonia reported an 11% incidence of plastic bronchitis, with IAV accounting for the majority of these cases (9.3%) (9).

*P. aeruginosa* is a Gram-negative *γ*-proteobacterium and an opportunistic pathogen capable of inducing severe infections, particularly in immunocompromised hosts. Its pathogenicity is mediated through pili and flagella, along with sophisticated virulence mechanisms including biofilm formation, quorum sensing, and secretion of various toxins such as exotoxins, proteases, and lipopolysaccharides (10, 11). While *P. aeruginosa* is a well-established pathogen in children with cystic fibrosis (CF), with initial acquisition occurring in 50% of patients by age 5.1 years (12), its role in respiratory infections among immunocompetent, non-CF children is less clearly defined. In the context of influenza-associated pneumonia, bacterial co-infection significantly increases disease severity (13). Among adults with community-acquired pneumonia secondary to influenza, the incidence of *P. aeruginosa* co-infection has been reported to be approximately 1.3% (14). In a pediatric cohort, the rate of P. aeruginosa co-infection with influenza was 3.4% (2/58) (15). However, population-level epidemiological data specifically characterizing the non-CF pediatric population remains scarce. Case reports have documented unusual presentations in healthy infants, including bloody pleural effusion (16), and *in vitro* studies have elucidated potential mechanisms facilitating secondary *P. aeruginosa* infection (17). IAV and *P. aeruginosa* co-infection resulting in obstructive mucopurulent cast formation in an immunocompetent, non-CF child is exceptionally rare. Herein, we present such a case.

## 2 Case report

A 13-month-old boy with a history of neonatal COVID-19 infection and no history of foreign body aspiration was transferred to our unit at 22:44 on December 3, 2023. He presented with a 1.5-day history of fever and cough, which progressed to hoarseness, barky cough and tachypnea over the preceding 6 h. Approximately 1.5 h prior to admission, he developed acute cyanosis and bradycardia with a heart rate dropping to 40 bpm. After 2 min of resuscitation, including supplemental oxygen, airway suctioning, and chest compressions, his color and heart rate normalized. He received dexamethasone (4 mg) and was transported to our hospital via ambulance on nasal cannula oxygen.

On admission, vital signs were: T 37 °C, HR 168 bpm, RR 39 bpm, BP 90/49 mmHg, oxygen saturation (SpO_2_) 90% on oxygen. The child appeared lethargic and cyanotic, with nasal flaring and marked intercostal, subcostal, and suprasternal retractions. On auscultation, bilateral coarse breath sounds were noted, accompanied by audible gurgling and inspiratory stridor.

**Figure 1:**
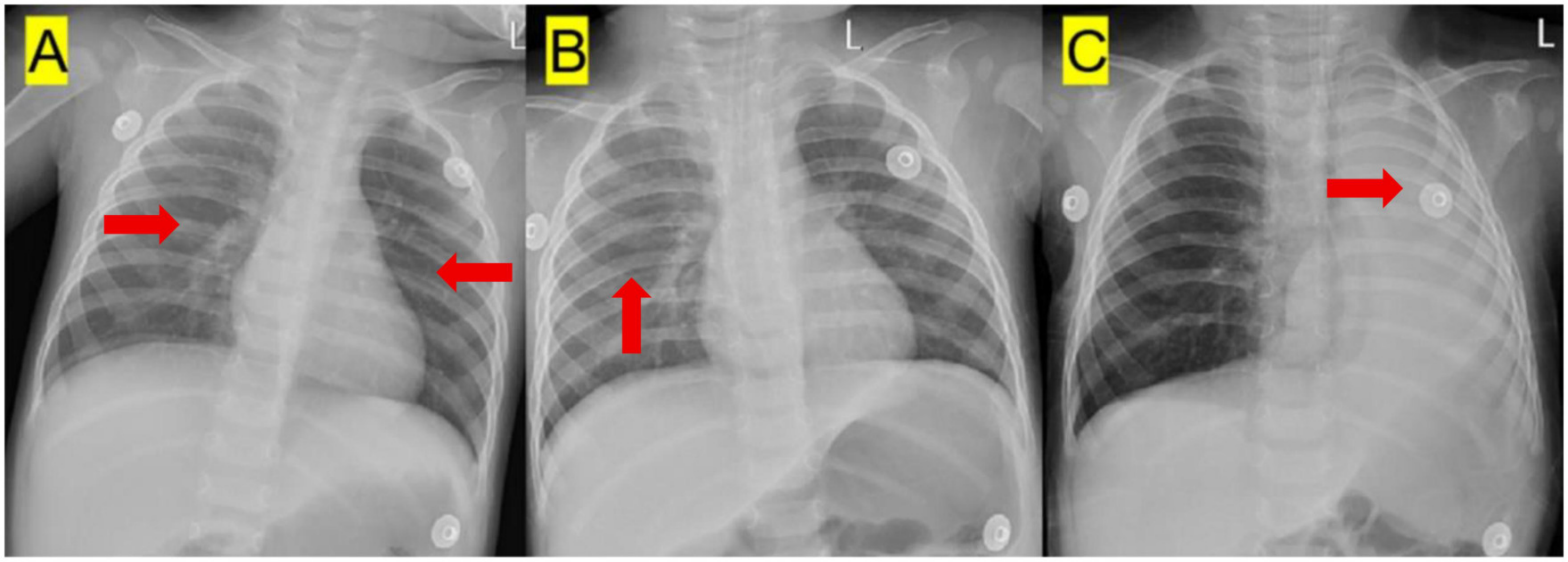
**(A)** Chest x-ray obtained at 23:42 (on admission) showing increased bronchovascular markings. **(B)** Chest x-ray obtained at 01:49 (2 h post-admission) showing increased radiopacity of the right lung. **(C)** Chest x-ray obtained at 06:24 (6 h and 40 min post-admission) showing complete left lung consolidation with collapse.

Due to the patient's critical condition and insufficient medical staff coverage during the night, transportation for a chest computed tomography (CT) scan was precluded. An on-site portable chest radiograph was obtained instead for initial assessment. Initial portable chest x-ray (23:42) showed bilateral bronchovascular markings (Figure 1A). Arterial blood gas [ABG, on 40% fraction of inspired oxygen (FiO_2_)] revealed pH 7.20, partial pressure of oxygen (PO_2_) 107 mmHg, partial pressure of carbon dioxide (SpO_2_) 36.8 mmHg. Labs showed white blood cell count (WBC) 8.55 × 10^9/L, neutrophils 66.2%, C-reactive protein (CRP) 28.89 mg/L, procalcitonin (PCT) 1.6 ng/mL, D-dimer 0.83 mg/L, and fibrin degradation products 2.7 mg/L.

He was diagnosed with acute laryngitis and grade IV laryngeal obstruction. Management initiated with nasal continuous positive airway pressure [FiO_2_ 40%, positive end-expiratory pressure, [PEEP] 4 cmH_2_O, respiratory rate [RR] 30 bpm, pressure control [PC] above 12 cmH_2_O], nebulized budesonide, and systemic methylprednisolone. His respiratory status deteriorated progressively, requiring an increase in FiO_2_ to 70%. Despite this, his SpO_2_ dropped to 80%, necessitating endotracheal intubation at 01:19 Ventilator settings were initially pressure-controlled [FiO_2_ 70%, PEEP 6 cmH_2_O, RR 30 bpm, PC above PEEP 10 cmH_2_O, tidal volume(Vt) ∼70 mL]. A chest x-ray at 01:49 confirmed the endotracheal tube [ETT]tip at T4 and increased opacity in the right lung (Figure 1B). The tube depth was adjusted.

A subsequent ABG (04:24, on 70% FiO_2_) showed pH 7.353, PO_2_ 74.1 mmHg, PCO_2_ 60.7 mmHg, prompting endotracheal suctioning and ventilator adjustments (PC above PEEP to 11 cmH_2_O, RR to 38 bpm). By 06:00, respiratory distress persisted with SpO_2_ at 85% and diminished breath sounds over the left lung. A repeat ABG (on 100% FiO_2_) showed severe hypoxia: pH 7.364, PO_2_ 42.8 mmHg, PCO_2_ 38.7 mmHg. A chest x-ray at 06:24 revealed complete left lung opacification with mediastinal shift and right lung hyperinflation (Figure 1C).

**Figure 2:**
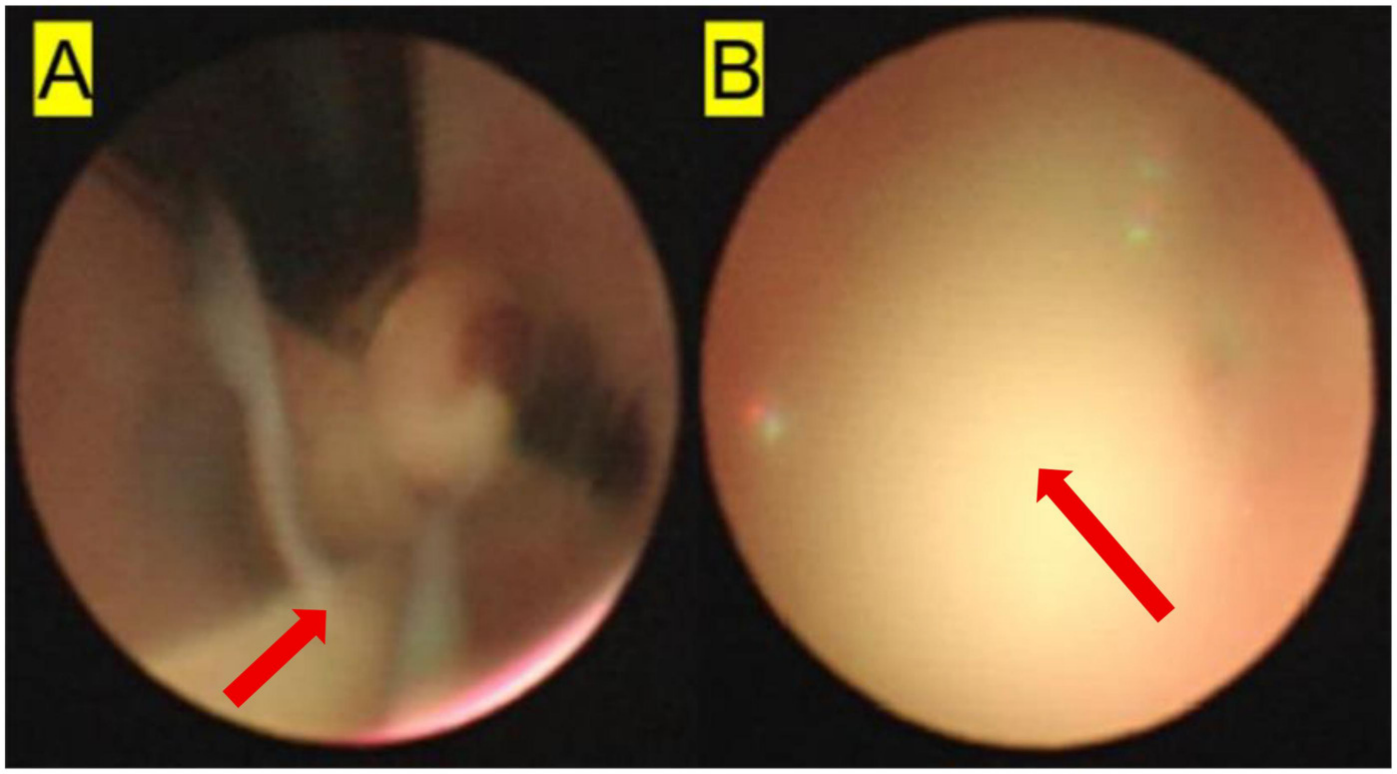
**(A)** Bronchoscopy revealed a mucus plug located at the carina. **(B)** Bronchoscopy revealed complete occlusion of the left main bronchus by cast-like, tenacious, yellow mucopurulent plugs.

**Figure 3:**
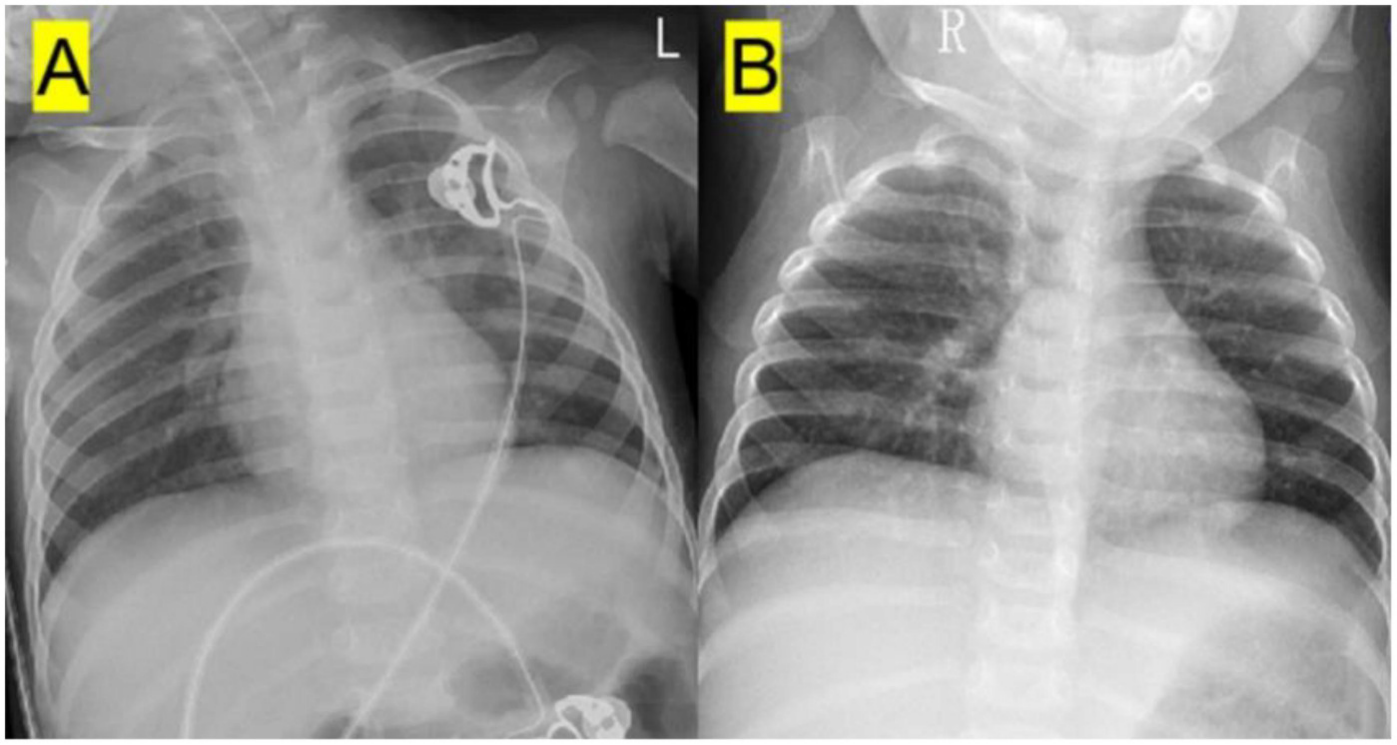
**(A)** Chest x-ray obtained 9 h after bronchoscopy showing successful left lung re-expansion. **(B)** Chest x-ray obtained 7 days after bronchoscopy (at discharge) showing resolution of pneumonia.

Emergency bronchoscopy was performed at 07:46, revealing copious tenacious secretions at the carina and complete occlusion of the left main bronchus by a mucus plug (Figures 2A,B). The plug was successfully removed, resulting in immediate re-expansion of the left lung on follow-up x-ray (Figure 3A). Post-procedure labs, obtained 10 h after admission, showed WBC 3,83 × 10^9^/L, neutrophils 59.9%, CRP 32.53 mg/L, and PCT 7.01 ng/mL. Antibiotic therapy was initiated with vancomycin (10 mg/kg/dose, q6 h) and cefoperazone-sulbactam (66.7 mg/kg/dose, q12 h).

At 15:52, a nasopharyngeal swab nucleic acid test returned positive for IAV. Peramivir (10 mg/kg.d) was added. The immunological results are shown in Table 1. Echocardiography revealed a 1 mm patent foramen ovale with no evidence of pulmonary hypertension or right-to-left shunt, and this finding was considered hemodynamically insignificant.

**Table 1:** Immunological laboratory findings.

Parameter	Patient Value	Normal Reference Range (boy,13 month-old)^a^
Complement		
C3	0.791 g/L	0.8–1.6 g/L
C4	0.21 g/L	0.16–0.48 g/L
Lymphocyte subsets		
CD4⁺ T cells	29.33% (372 cells/μL)	24.08%–42.52% (902–2,253 cells/μL)
CD8⁺ T cells	20.54% (260 cells/μL)	19%–32.15% (580–1,735 cells/μL)
CD4/CD8 Ratio	1.43	0.9–2.13
NK cells	6.2% (79 cells/μL)	7.21%–20.9% (270–1,053 cells/μL)
B Cells	42.05% (533 cells/μL)	13.23%–26.39% (461–1,456 cells/μL)
Immunoglobulins		
IgG	3.7 g/L	5.09–10.09 g/L
IgA	0.208 g/L	0.31–0.67 g/L
IgM	0.298 g/L	0.98–1.78 g/L

C3, complement component 3; C4, complement component 4; CD4⁺ T cells, cluster of differentiation 4 positive T lymphocytes; CD8⁺ T cells, cluster of differentiation 8 positive T lymphocytes; NK cells, natural killer cells; B Cells, B lymphocytes; IgG, immunoglobulin G; IgA, immunoglobulin A; IgM, immunoglobulin M.

a Normal reference ranges are derived from age-matched healthy Chinese children based on *Zhu Futang Practice of Pediatrics* (9th edition).

On day 3, targeted Next-Generation Sequencing (tNGS) of bronchoalveolar lavage fluid (BALF) confirmed IAV (H3N2 normalized count 607,085, estimated concentration 10^6^ copies) and *P. aeruginosa* (normalized count 221,477, estimated concentration 107 copies). BALF and sputum cultures grew *P. aeruginosa*. Vancomycin was discontinued in light of susceptibility results. The blood culture was negative. He was successfully extubated and weaned from mechanical ventilation on Day 4. He completed a 5-day course of peramivir and an 8-day course of cefoperazone-sulbactam, with excellent adherence and no reported adverse effects. His condition stabilized steadily, with significant improvement on follow-up chest x-ray (Figure 3B). He was discharged on hospital day 9.

## 3 Discussion

Acute croup is a common pediatric emergency, often triggered by viral infections. This case first vividly illustrates a dynamic progression of IAVco-infection with *P. aeruginosa*. It began with typical croup symptoms, followed by rapid spread of inflammation from the upper to the lower airways, culminating in respiratory failure and left lung atelectasis due to mechanical obstruction. This case highlights that rapidly progressive respiratory distress warns of central airway mechanical obstruction. The bronchoscopic finding of mucus plugs highlighted the importance of purulent casts as a reversible cause of critical airway obstruction in this setting. In cases of steroid-unresponsive, rapidly progressive croup, the possibility of severe IAV coinfection with *P. aeruginosa* must be considered. This pathogen synergy can cause necrotizing bronchiolitis with obstructive plug formation, necessitating urgent microbiological investigation and bronchoscopic intervention.

The presence of mucopurulent casts necessitates consideration of underlying conditions such as severe asthma, CF, and allergic bronchopulmonary aspergillosis (ABPA). In this case, the patient had no history of recurrent cough, sputum production, or wheezing; no personal or family history of atopy (eczema or allergic rhinitis); and no family history of asthma in first- or second-degree relatives. During hospitalization, physical examination revealed no wheezing on lung auscultation. Serial complete blood counts showed normal eosinophil proportions (0%–0.2% of total leukocytes, absolute count 0–0.01 × 10⁹/L). BALF was negative for Aspergillus by tNGS. Follow-up revealed no recurrent respiratory symptoms. These clinical findings are inconsistent with severe asthma, CF, or ABPA. However, sweat chloride testing and cystic fibrosis transmembrane conductance regulator genetic analysis were not performed, and long-term follow-up is warranted to definitively exclude latent predisposing conditions.

*P. aeruginosa* is not a common bacterial pathogen isolated from patients with influenza. The pathogenesis of severe bacterial infections secondary to influenza mainly involves the disruption of the respiratory epithelial barrier, dysfunction of immune cells, and excessive immunopathological damage (18). Infection with influenza impairs the tight junctions of respiratory epithelial cells, reduces the production of antimicrobial peptides, and upregulates the expression of bacterial adhesion receptors (19, 20). Consequently, it weakens the physical and biochemical defense barriers of the respiratory tract, thereby creating favorable conditions for bacterial invasion and colonization. Meanwhile, influenza infection impairs the functions of alveolar macrophages, *γδ* T cells, invariant natural killer T cells, and dendritic cells, compromising their antibacterial activity and immune activation capacity (21). In addition, influenza infection impairs the phagocytic and bactericidal abilities of neutrophils, and promotes the polarization of macrophages toward the M2 phenotype, which inhibits their antibacterial functions. In the setting of coinfection, influenza and bacterial components synergistically activate the complement system and pattern recognition receptors, leading to excessive inflammatory responses, pyroptosis, and necrosis. These processes release a large number of inflammatory mediators, exacerbate tissue damage, and ultimately result in severe immunopathological injury and acute lung injury (17, 22, 23). Notably, in this pediatric case, the observed laboratory findings—specifically, decreased levels of complement C3, CD3+, CD4+ T cells, and natural killer cells (NK cells)—align closely with the described immunopathogenic mechanisms (Table 1).

Reflecting on our initial management, the mild elevation of inflammatory markers (CRP only 28.89 mg/L) was attributed to the stress response, and the classic croup presentation led to a presumptive diagnosis of isolated viral infection. This interpretation unfortunately resulted in a delay in initiating empiric antibacterial therapy. This case, however, offers clinically actionable insights for managing severe pediatric respiratory infections with rapid deterioration. First, steroid-resistant croup with rapid progression to respiratory failure and unilateral lung collapse should immediately raise suspicion for central airway obstruction, prompting emergent bronchoscopy. Second, although *P. aeruginosa* is uncommon in immunocompetent children, it must be considered in severe influenza-associated pneumonia when the clinical course deviates from typical viral illness. Third, dynamic PCT monitoring—rather than a single measurement—can provide early warning of evolving bacterial co-infection, as illustrated by the sharp PCT rise over 10 h despite only modest CRP elevation. Fourth, bronchoscopy serves a dual purpose here: it is both a life-saving therapeutic intervention for mechanical obstruction and a diagnostic tool for precise pathogen identification through lower respiratory tract sampling.

Several limitations of this report warrant acknowledgment. Although next-generation sequencing of BALF identified the IAV as H3N2, subclade-specific characterization was not performed. Given that particular H3N2 subclades (e.g., subclade K) are known to be more virulent, we recognize the value of such analysis and plan to incorporate subclade typing in future cases of severe influenza presenting with unusual complications. Furthermore, as a single case report, our findings have limited generalizability to the broader pediatric population; additional case series are therefore warranted.

## 4 Conclution

This case describes a life-threatening pediatric emergency: IAV and *P. aeruginosa* co-infection presenting as steroid-resistant croup with obstructive mucopurulent cast formation, leading to rapid respiratory failure and unilateral lung collapse. It underscores that when common presentations deviate from their expected course, clinicians must broaden their diagnostic framework. Early recognition of atypical features and timely bronchoscopic intervention can be life-saving.

## Data Availability

The original contributions presented in the study are publicly available. This data can be found here: The genetic and genomic sequence data have been deposited and are now publicly available in NCBI SRA. Accession: PRJNA1472379, link: https://www.ncbi.nlm.nih.gov/sra/PRJNA1472379.
